# A new hourly dataset for photovoltaic energy production for the continental USA

**DOI:** 10.1016/j.dib.2022.107824

**Published:** 2022-01-13

**Authors:** Weiming Hu, Guido Cervone, Andre Merzky, Matteo Turilli, Shantenu Jha

**Affiliations:** a Scripps Institution of Oceanography, University of California, San Diego, United States; b Institute for Computational and Data Sciences, The Pennsylvania State University, United States; c Earth and Environmental Systems Institute, The Pennsylvania State University, United States; d Research Application Laboratory, National Center for Atmospheric Research, United States; e Department of Electrical and Computer Engineering, Rutgers University, United States

**Keywords:** Renewable energy, Solar photovoltaic, Ensemble simulation, Distributed computing

## Abstract

This new dataset is an ensemble of solar photovoltaic energy production simulations over the continental US. The simulations are carried out in three steps. First, a weather forecast system is used for the predictions of incoming insolation; then, forecast ensembles with 21 members are generated using the Analog Ensemble technique; finally, each ensemble member is used to simulate 13 different solar panels. In total, there are 21×13=273 simulated scenarios. Simulations are carried out for the entire year 2019, with a temporal resolution of one hour, and a spatial resolution of 12 km. The data provide a high spatio-temporal analysis of the power production under different weather and engineering scenarios. The size of the entire dataset is about 1 TB but can be openly accessed by days and scenarios. Details on how to access and use such a dataset are provided in this article.

## Specifications Table

**Table:** 

Subject	Renewable Energy, Sustainability and the Environment
Specific subject area	Ensemble simulation of solar photovoltaic energy production to study generation efficiency and forecast uncertainty
Type of data	NetCDF
	Table
How data were acquired	The raw data are collected from the operational weather forecast system, NAM-NMM.
Data format	Simulated
Parameters for data collection	The weather forecast system is NAM-NMM (hereafter NAM) with a 12 km horizontal resolution. Operational forecasts are collected for 2019. Parallel Analog Ensemble has version 4.4.3, pvlib has version 0.8.1, and EnTK has version 1.6.7.
Description of data collection	This article introduces a secondary dataset that is simulated from a multi-step workflow, including a weather forecast system, an ensemble generation, and a power system simulation.
Data source location	Primary data source from NCEP: https://www.ncei.noaa.gov/products/weather-climate-models/north-american-mesoscale The Pennsylvania State University
Data accessibility	Scholar Sphere: https://scholarsphere.psu.edu/resources/dacba268-d084-4e0e-a674-670217c59891

## Value of the Data

• These new data provide an ensemble of power production simulations with high spatial and temporal resolutions. They can be used for a multitude of studies, from assessing the performance of forecasting systems, to identify the best locations for solar power generation. Simulations are generated with multiple solar panels, thus together representing a valid and comprehensive tool for studying power generation efficiency and simulation uncertainty.
• Institutions and researchers involved in renewable energy, specifically in the photovoltaic solar sector, can benefit from these data because it provides simulations for a wide range of weather and engineering scenarios. Weather and economic modelers can also benefit from this study by deriving uncertainty from the ensemble simulations.
• The simulated power production data can be used in a variety of applications including power potential assessment at a specific location. The simulation ensemble enables research on forecast uncertainty quantification which is critical for grid operation and renewable penetration.
• The additional value of these data lies in its readiness and completeness. Generation of the data is computationally intensive but this dataset enables rapid assessment of solar power generation with various weather scenarios and panel configurations.

## 1 Data Description

This dataset contains hourly power production simulation for 2019 over the Continental US (CONUS) with a 12 km spatial resolution. There are 21 members in the weather forecast ensemble and 13 solar panel modules. In total, there are year-round power simulations for 273 different scenarios considering weather and engineering conditions. Considering the high spatial and temporal resolution of the simulation, the entire dataset is about 1 TB in size but can be accessed by days and scenarios, making each file about 246 MB.

**Fig. 1:**
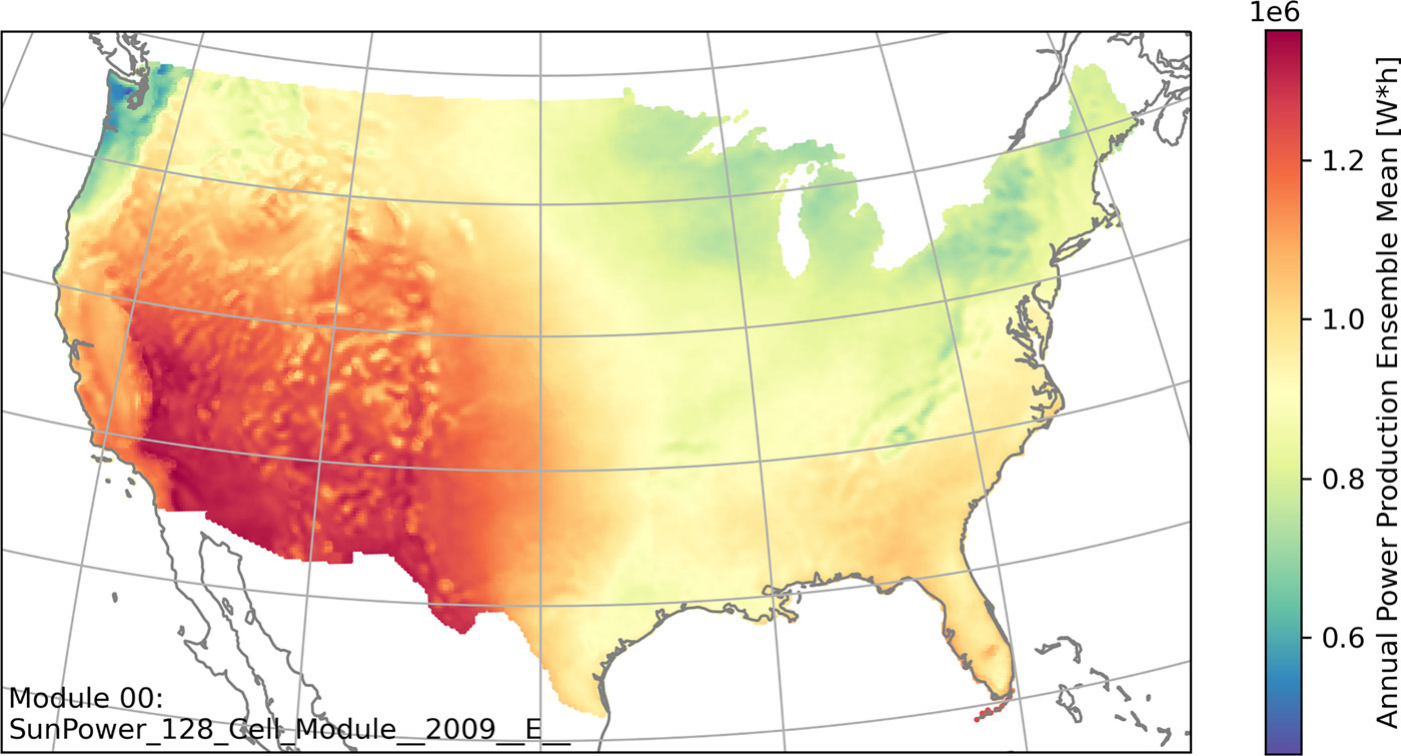
Annual power production calculated from ensemble mean for 2019 with the solar panel module, SP128.

Fig. 1 shows the annual accumulated power production calculated from ensemble mean for 2019. The visualization is created for the solar panel module, SP128. The domain of interest is the CONUS which is covered by a 12 km mesh grid with 56,776 grid points. It is typical to expect a high amount of power production from photovoltaic solar in western and southwestern US. The Pacific Northwest shows a lower production especially over the coastal region. This is related to the high amount of cloud cover year-round.

**Fig. 2:**
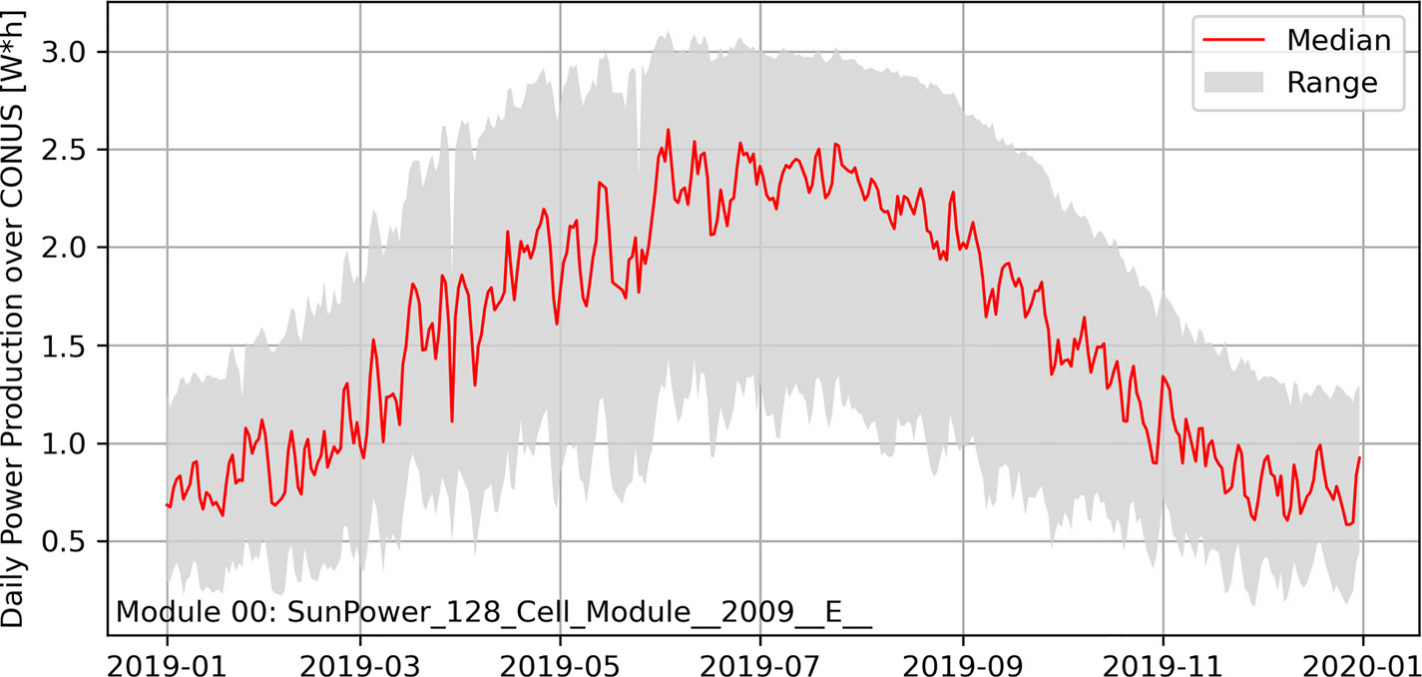
Power production ensemble for 2019 with the solar panel module, SP128, calculated across CONUS. The range of the 21 forecast members (weather scenarios) is shown in the grey shade and the median member is shown in the solid red line. (For interpretation of the references to color in this figure legend, the reader is referred to the web version of this article.)

Fig. 2 shows the hourly power production time series for 2019 with the solar panel module, SP128, accumulated over CONUS. There are 21 forecast members (weather scenarios); the range of the ensemble is shown in the grey shade and ensemble median is shown in the solid red line. Considering the wide range of possible atmospheric states, the variability of the simulated power production is significant, especially entering the spring and the summer seasons. During the summer season, the upper bound of the power production reaches a plateau and the ensemble median approaches the upper bound. This is because solar panels are already performing at its maximum power level under excessive solar irradiance condition.

Figs. 1 and 2 demonstrate only one out of the 13 simulated modules. All other modules can be analyzed similarly. While the general spatial patterns and temporal trends of power production should remain similar, the predictability of each module varies depending on the atmospheric condition and the condition of the solar panel module. This dataset provides a useful tool for the analysis of power production predictability.

**Fig. 3:**
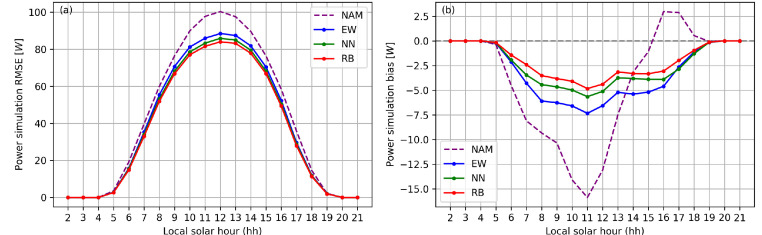
RMSE (a) and bias (b) of PV power simulation ensemble mean per square meter by forecast lead times for 2019. NAM represents the baseline model that is used as input; Equal Weighting (EW), Nearest Neighbor (NN), and Region Based (RB) optimizations have been compared when applying the AnEn method. The final dataset is generated with the RB optimization method [1].

To quantify the quality of the dataset, Fig. 3 shows the Root Mean Square Error (RMSE) and bias for the photovoltaic power simulation ensemble mean. Results are averaged across the CONUS as a function of local time. Three optimization methods for the Analog Ensemble (AnEn) parameters have been tested and the verification results are included to compare with the baseline model, North American Mesoscale Model (NAM), and the parameters optimized from the Region Based method were used to generate the final dataset. Details on the experiments and the optimization can be found in Hu et al. [1]. At the peak hour (12 h) of solar irradiance, RB optimized AnEn simulation reduces the RMSE from 100 to 83.99 $\text{W}/{\text{m}}^{2}$, equivalent to a 16.26% reduction compared to the baseline model. At the previous hour (11 h), model bias is reduced from −15.87 $\text{W}/{\text{m}}^{2}$ to −4.83 $\text{W}/{\text{m}}^{2}$, although the magnitude of change is much smaller than that of the RMSE.

The dataset is hosted on a publicly available repository. It contains the following files:

1. *README.md*: A summary file of the data repository
2. *modules.csv*: A CSV file that contains detailed information for each simulated photovoltaic module
3. *coordinates.nc*: An NetCDF file that contains coordinates of the simulated locations over the continental US.
4. *Example.ipynb*: An example Jupyter notebook written in python that demonstrates how to interact with the data from this repository
5. *Example.html*: The HTML file generated from *Example.ipynb*
6. *modules_[ID]*: There are 13 simulated modules and the ID corresponds to the modules listed in *modules.csv*. Each module folder contains daily simulation files stored in NetCDF. File names follow the convention *YYYYMMDD_module_[ID]_analog.nc*. More information on how to interact with the data files can be found in *Example.ipynb*.

## 2 Experimental Design, Materials and Methods

**Fig. 4:**
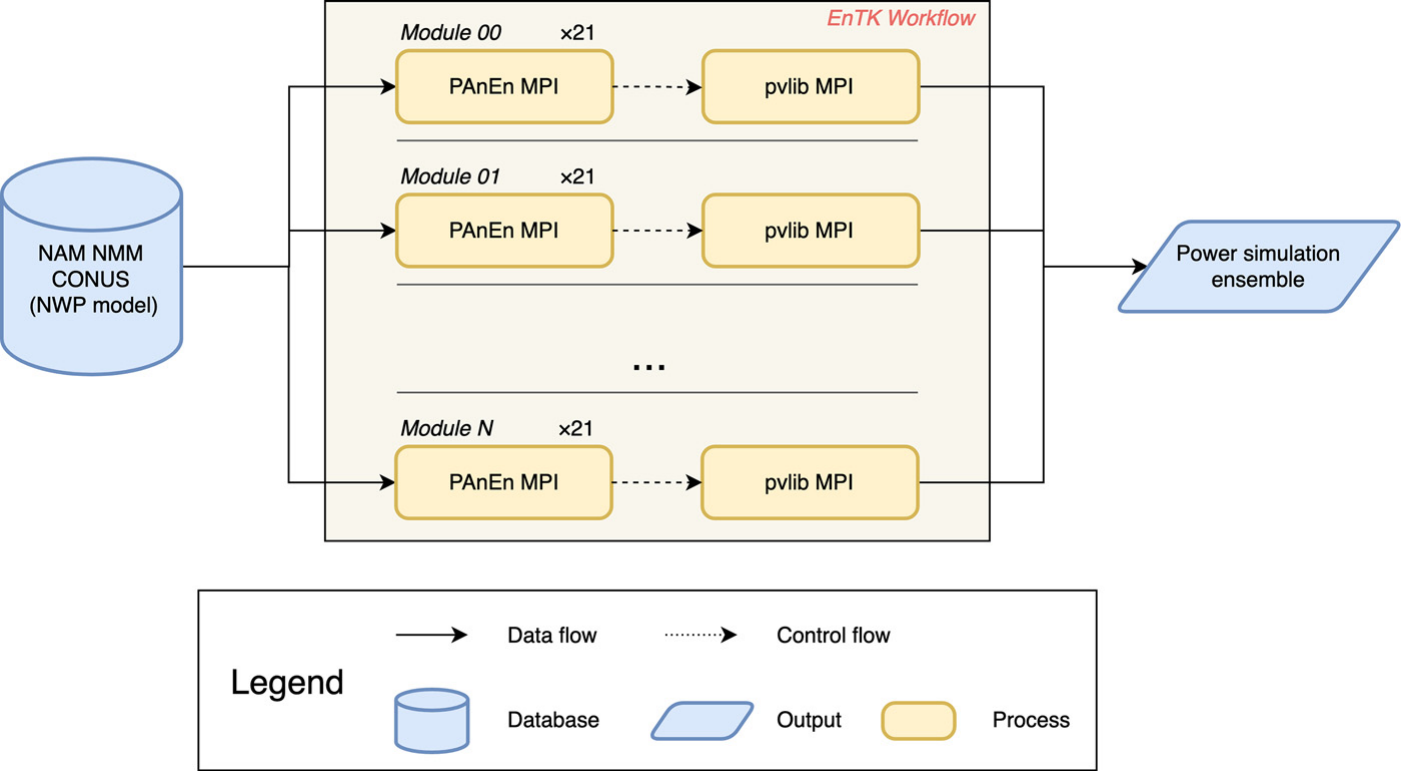
Workflow diagram of the dataset generation.

The dataset is generated from a three-stage workflow. Fig. 4 shows the workflow of generating the simulation dataset with three components, the weather forecast system, the AnEn generation [2], and the power production simulation.

*Weather Model* A weather forecast system provides the primary forcing to simulate power production including estimates of solar irradiance, wind speed, and temperature. The model used in this dataset is the NAM forecast system [3]. It is a deterministic weather model that is run four times a day and provides hourly forecasts up until 84 h into the future for each run. In this dataset, the model run at 00z is collected and the lead times between 006 h and 032 h are used to cover the day-ahead weather forecasts over CONUS. Forecasts for 2019 are collected. However, there are six days missing due to the operational model malfunctioning. These missing dates are 2019-02-04, 2019-05-18, 2019-05-19, 2019-09-03, 2019-11-09, and 2019-11-10. Therefore, there are in total 359 valid days throughout the year.

*Ensemble Generation* NAM is a deterministic weather model which only provides a single state of the atmosphere. An additional process is carried out to generate the weather ensembles using the Parallel Analog Ensemble library [4], [5]. AnEn is a technique to generate accurate and calibrated forecast ensembles from deterministic weather forecasts without running the model multiple times. Therefore, it is well suited for operational forecasts where ensembles can be directly generated when the operational model is run. AnEn looks for similar historical weather forecasts and then use the corresponding historical observations as the ensemble members. The set of predictors used by the AnEn include downward shortwave radiation flux, surface pressure, temperature at 2  m above ground, total cloud cover, wind speed, and wind direction. These parameters are pre-selected because they have relatively high correction with the final solar energy production and they have been found to be useful predictors for solar energy forecasts [6], [7]. The search period is 2017 and 2018, and 21 ensemble members are generated to account for sampling error [8]. Please refer the [2], [4] for a detailed description of the AnEn and the open-source software used.

*Power Simulation* Finally, weather forecasts are fed into a simulated power system to generate the power production estimates at each grid point. This process is carried out with the *pvlib* python package [9]. *pvlib* is an open-source and community-supported tool that simulate the performance of photovoltaic energy systems. A 10 KW system is assumed at each model grid cell (12 km by 12 km) and the number of panels is therefore calculated as the desired system output divided by the nominal power output of a single panel. Global horizontal irradiance, air temperature, and wind speed are input to the power system. Global horizontal irradiance is used to estimate the incident irradiance reaching the panels. Air temperature and wind speed are used to estimate the cell temperature that could cause an impact on panel efficiency. The decomposition of global horizontal irradiance is carried out using the DISC model [10]. Hay & Davies’s 1980 model [11], [12] is used to calculate the diffuse irradiance from the sky. Cell temperature is estimated per the Sandia Array Performance Model [13], and finally, the power output is estimated using the SAPM model [13].

All together, the three components are built together with the RADICAL Ensemble Toolkit (EnTK) [14]. EnTK is a python library for developing and executing large-scale ensemble-based workflows. It allows a high level of flexibility while defining the workflow and simplifies the process of managing communication and jobs on a cluster. This dataset was generated on the supercomputer, Cheyenne, from the National Center of Atmospheric Research. The execution of the entire workflow costs 18,675 core*hours.

It is, however, possible to apply the same workflow on a historical time series of data collected on site. For example, the Surface Radiation Budget Network (SURFRAD) [15], established in 1993 through the support of NOAA’s Office of Global Programs, aims to provide high-quality ground measurements of variables related to energy budget (e.g. global horizontal irradiance) and weather conditions (e.g. air temperature and wind speed). Another option is to use the historical weather records from the Automated Surface Observing System (ASOS), also maintained by NOAA. The key consideration, for this particular dataset, of using a weather forecast system is twofold, a complete temporal and spatial coverage and the ability to study the uncertainty of solar photovoltaic energy forecast. SURFRAD has eight stations and ASOS has around 700 stations across the CONUS, which only provide distributed local coverage. A weather forecast ensemble also provides a series of possible atmospheric states to test how sensitive the performance simulation is to forecast uncertainty.

Table 1 lists out the 13 solar panel modules simulated in the dataset with detailed information including the manufacturer, the year, and the panel efficiency. The modules are selected from the Sandia Module Database [9]. The database contains 523 modeled solar panel modules but most of the modules share similar characteristics in size and power efficiency. We selected a subset of the modules by running a hierarchical clustering algorithm based on *Area, Cells_in_Series*, and *MP* (Maximum Power). We also excluded modules prior to 2008 to prefer recent technological advances.

**Table 1:** Information table for the 13 simulated solar panel modules. **STC** stands for power production under the standard testing condition, a cell temperature of 25 ${}^{\circ }$C and a solar irradiance of 1000 W/m${}^{2}$.

ID	Manufacturer	Code	Area [${\text{m}}^{2}$]	Material	Year	STC [W]	Efficiency
0	SunPower	SP128	2.144	c-Si	2009	400	0.187
1	First Solar	FS272	0.72	CdTe	2009	72.5	0.1007
2	Solar Frontier	SF160S	1.22	CIS	2013	160	0.132
3	SolFocus	SF1100S	1.502	GaAs	2010	415	0.284
4	Kyocera	KD135GX	1.002	mc-Si	2008	135	0.1347
5	Kyocera	KS20	0.072	mc-Si	2008	20	0.280
6	Kyocera	KC85T	0.656	mc-Si	2008	85	0.134
7	Suniva	ST240	1.643	c-Si	2009	240	0.147
8	BP Solar	BP3232G	1.6068	c-Si	2010	230	0.148
9	SunPower	SP305	1.63	c-Si	2008	305	0.187
10	LG	LG290N1C	1.64	c-Si	2013	290	0.179
11	Sharp	ND216U1F	2.63	mc-Si	2008	215	0.083
12	Silevo	STU300	1.68	c-Si	2014	300	0.178

## 3 Data Access

The data repository offers open access via Penn State Scholar Sphere available at https://scholarsphere.psu.edu/resources/dacba268-d084-4e0e-a674-670217c59891.

An example of how to interact with and visualize the dataset can be found in *Example.ipynb*, available from the same data repository. Potential data users are encouraged to review the generated HTML page from the *Example.ipynb* directly from the repository.

## Ethics Statement

This work is supported by the EarthCube office, US National Science Foundation. Information on the funded project is available at https://www.nsf.gov/awardsearch/showAward?AWD_ID=1639707.

## CRediT authorship contribution statement

**Weiming Hu:** Conceptualization, Methodology, Software, Formal analysis, Data curation, Writing – original draft. **Guido Cervone:** Conceptualization, Methodology, Writing – review & editing, Supervision. **Andre Merzky:** Resources, Software, Writing – review & editing. **Matteo Turilli:** Resources, Software, Writing – review & editing. **Shantenu Jha:** Resources, Software, Writing – review & editing.

## Declaration of Competing Interest

The authors declare that they have no known competing financial interests or personal relationships which have, or could be perceived to have, influenced the work reported in this article.
